# Publisher Correction: Targeting of NAT10 enhances healthspan in a mouse model of human accelerated aging syndrome

**DOI:** 10.1038/s41467-026-69133-5

**Published:** 2026-03-18

**Authors:** Gabriel Balmus, Delphine Larrieu, Ana C. Barros, Casey Collins, Monica Abrudan, Mukerrem Demir, Nicola J. Geisler, Christopher J. Lelliott, Jacqueline K. White, Natasha A. Karp, James Atkinson, Andrea Kirton, Matt Jacobsen, Dean Clift, Raphael Rodriguez, Sanger Mouse Genetics Project, Carl Shannon, Mark Sanderson, Amy Gates, Joshua Dench, Valerie Vancollie, Catherine McCarthy, Selina Pearson, Emma Cambridge, Christopher Isherwood, Heather Wilson, Evelyn Grau, Antonella Galli, Yvette E. Hooks, Catherine L. Tudor, Angela L. Green, Fiona L. Kussy, Elizabeth J. Tuck, Emma J. Siragher, Robbie S. B. McLaren, Agnieszka Swiatkowska, Susana S. Caetano, Cecilia Icoresi Mazzeo, Monika H. Dabrowska, Simon A. Maguire, David T. Lafont, Lauren F. E. Anthony, Maksymilian T. Sumowski, James Bussell, Caroline Sinclair, Ellen Brown, Brendan Doe, Hannah Wardle-Jones, Nicola Griggs, Mike Woods, Helen Kundi, George McConnell, Joanne Doran, Mark N. D. Griffiths, Christian Kipp, Simon A. Holroyd, David J. Gannon, Rafael Alcantara, Ramiro Ramirez–Solis, Joanna Bottomley, Catherine Ingle, Victoria Ross, Daniel Barrett, Debarati Sethi, Diane Gleeson, Jonathan Burvill, Radka Platte, Edward Ryder, Elodie Sins, Evelina Miklejewska, Dominique Von Schiller, Graham Duddy, Jana Urbanova, Katharina Boroviak, Maria Imran, Shalini Kamu Reddy, David J. Adams, Stephen P. Jackson

**Affiliations:** 1 The Wellcome Trust/Cancer Research UK Gurdon Institute and Department of Biochemistry, University of Cambridge, Cambridge, CB2 1QN UK; 2 The Wellcome Trust Sanger Institute, Hinxton, Cambridge, CB10 1SA UK; 3 Discovery Sciences, IMED Biotech Unit, AstraZeneca, Cambridge, CB4 0WG UK; 4 Drug Safety and Metabolism, IMED Biotech Unit, AstraZeneca, Cambridge, CB2 23AT UK; 5 Laboratory of Molecular Biology, Cambridge, CB2 OQH UK; 6 Institut Curie, PSL Research University, Paris, Cedex 05 France; 7 CNRS UMR3666, 75005 Paris, France; 8 INSERM U1143, 75005 Paris, France; 9 Present Address: Department of Clinical Biochemistry, Cambridge Institute for Medical Research, University of Cambridge, Cambridge, CB2 0XY UK

Correction to: *Nature Communications* 10.1038/s41467-018-03770-3, published online 27 April 2018

In the version of this article initially published, in the graph in Fig. 3a, the right-hand bars labelled “*Nat10*^+/–^” should have read “*Nat10*^–/–^.” Due to the age of the article, the figure cannot be updated directly. A correct version of Fig. 3a is available as Supplementary information accompanying this amendment. This notice serves to correct the article.

## Supplementary information

Correct Fig. 3.

