# Supplementary material for: Targeting of NAT10 enhances healthspan in a mouse model of human accelerated aging syndrome
Source: Nat Commun. 2018 Apr 27;9:1700. doi: 10.1038/s41467-018-03770-3 (PMC5923383; doi:10.1038/s41467-018-03770-3)
Supplement: Supplementary file 1 — Supplementary Information [file 41467_2018_3770_MOESM1_ESM.pdf]

Supplementary Materials for

Targeting of NAT10 enhances healthspan in a mouse model of human accelerated aging syndrome

Balmus, Larrieu et al.

Targeting of NAT10 enhances healthspan in a mouse model of human accelerated aging syndrome

Gabriel Balmus[§1,2], Delphine Larrieu[§*1,3], Ana C Barros[1,2], Casey Collins[2], Monica Abrudan[2], Mukerrem Demir[1], Nicola J Geisler[1,2], Christopher J. Lelliott[2], Jacqueline K. White[2], Natasha A Karp[2,4], James Atkinson[5], Andrea Kirton[2], Matt Jacobsen[5], Dean Clift[6], Raphael Rodriguez[7,8,9], Sanger Mouse Genetics Project[Ψ], David J Adams[2], Stephen P Jackson[*1]

[§] These authors contributed equally to the work

* Correspondence to s.jackson@gurdon.cam.ac.uk and dl437@cam.ac.uk

[Ψ] A full list of consortium members appears at the end of the paper

1. The Wellcome Trust/Cancer Research UK Gurdon Institute and Department of Biochemistry, University of Cambridge, CB2 1QN, United Kingdom.

2. The Wellcome Trust Sanger Institute, Hinxton, Cambridge, CB10 1SA, United Kingdom.

3. Current address: Cambridge Institute for Medical Research, Department of clinical biochemistry, University of Cambridge, CB2 0XY, United Kingdom.

4. Discovery Sciences, IMED Biotech Unit, AstraZeneca, Cambridge, CB4 0WG, UK

5. Drug Safety and Metabolism, IMED Biotech Unit, AstraZeneca, Cambridge, CB223AT, UK

6. Laboratory of Molecular Biology, Cambridge, CB2 OQH, United Kingdom.

7. Institut Curie, PSL Research University, Paris Cedex 05, France.

8. CNRS UMR3666, 75005 Paris, France.

9. INSERM U1143, 75005 Paris, France.

Supplementary file includes:

Supplementary Tables 1 to 4
Supplementary Figures 1 to 11

Other Supplementary Materials for this manuscript include the following:

Supplementary Data 1 to 5
Supplementary Movies 1 to 3

Supplementary figures

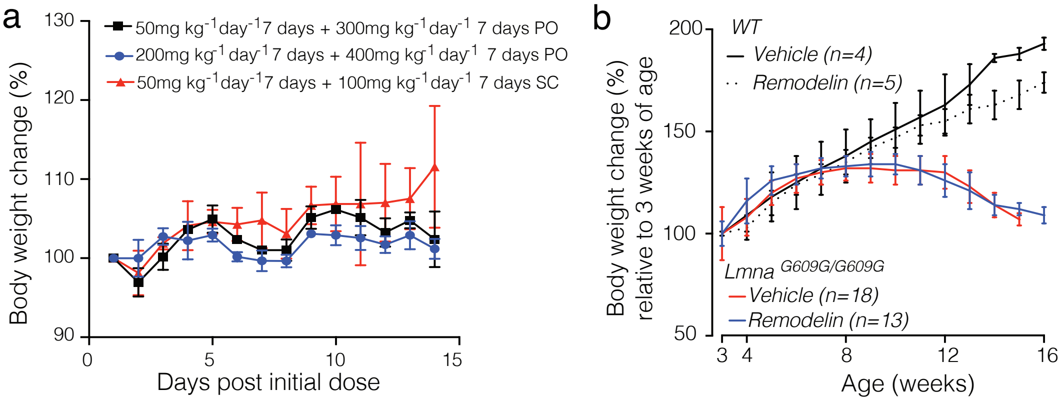

c

*Lmna $^{G609G/G609G}$ (n=3)	Remodelin (ng/g tissue)	Vehicle
Skeletal muscle	3689	<LLOQ
Liver	8563	<LLOQ
Brain	6284	<LLOQ

* 100mg per kg Remodelin daily for 6 weeks the tissues from n=3 mice were pooled;
Lower Limit of Quantification (LLOQ) = 5ng per g tissue

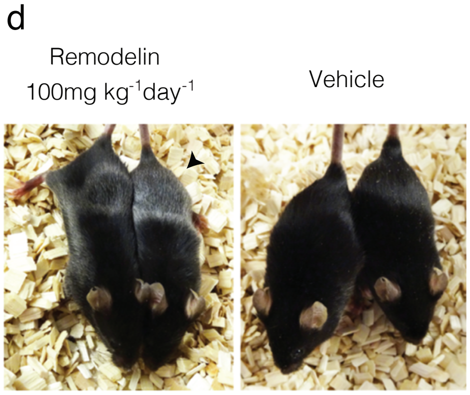

Supplementary Fig. 1: Assessment of Remodelin effects *in vivo*. a Toxicity assessment of Remodelin treatment on 6-week old C57BL/6N female mice. Group 1 (n=3) and 2 (n=3) were administered daily oral doses of the molecule (PO) as indicated, whereas group 3 (n=3) received Remodelin by subcutaneous injection (SC). No body weight loss or toxicity was observed on these regimes after 2 weeks of treatment. Error bars represent mean ± s.d. **b** Long-term treatment of the *Lmna*$^{G609G/G609G}$ mice with Remodelin (100mg per kg per day) showed no drug-dependent body weight loss. Error bars represent mean ± s.d. **c** Long term treatment with 100mg per kg Remodelin showed accumulation of the compound in tissues from *Lmna*^{G609G} mice. Plasma analysis revealed presence of the compound at 1 hour post treatment (ranging from 8.5 ng per ml to 30.5 ng per ml) and clearance by 24 hours post treatment in both WT and *Lmna*^{G609G} mice. **d** Hair graying was observed upon Remodelin treatment in all mice after several weeks' treatment in both WT (left mouse on each panel) and *Lmna*$^{G609G/G609G}$ mice (right mouse).

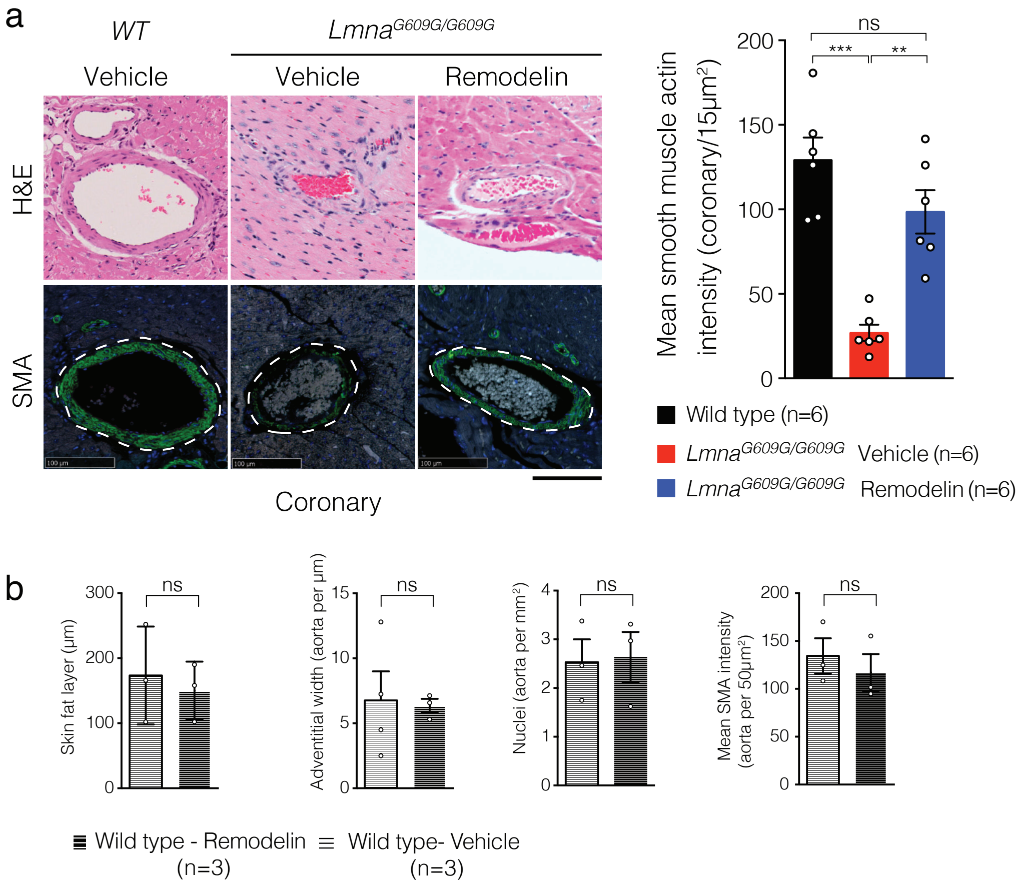

Supplementary Fig. 2: Remodelin improves the coronary pathology of HGPS mice. a Representative images of hematoxilin and eosin (H&E) and smooth muscle actin (SMA) staining of heart sections representing coronary arteries (left panels; size bar 100µm) showing improved integrity of the artery wall in terminal *Lmna*$^{G609G/G609G}$ mice treated with Remodelin. Right panel: bar graph of the quantification of the SMA mean intensity per 15µm^2 (mean ± s.d.; individual data points represented; ns = not significant; **p<0.01, ***p<0.001; two-tailed Student's t-test;). **b** Bar graph presenting the quantification of different indicated parameters in the aorta of the wild type (WT) Remodelin treated mice (n=3) vs. Vehicle treated mice (n=3). Remodelin has no significant effect (ns) as compared to Vehicle treatment and for simplicity in Figure 2 **a-d** the two have been pooled in one group (WT).

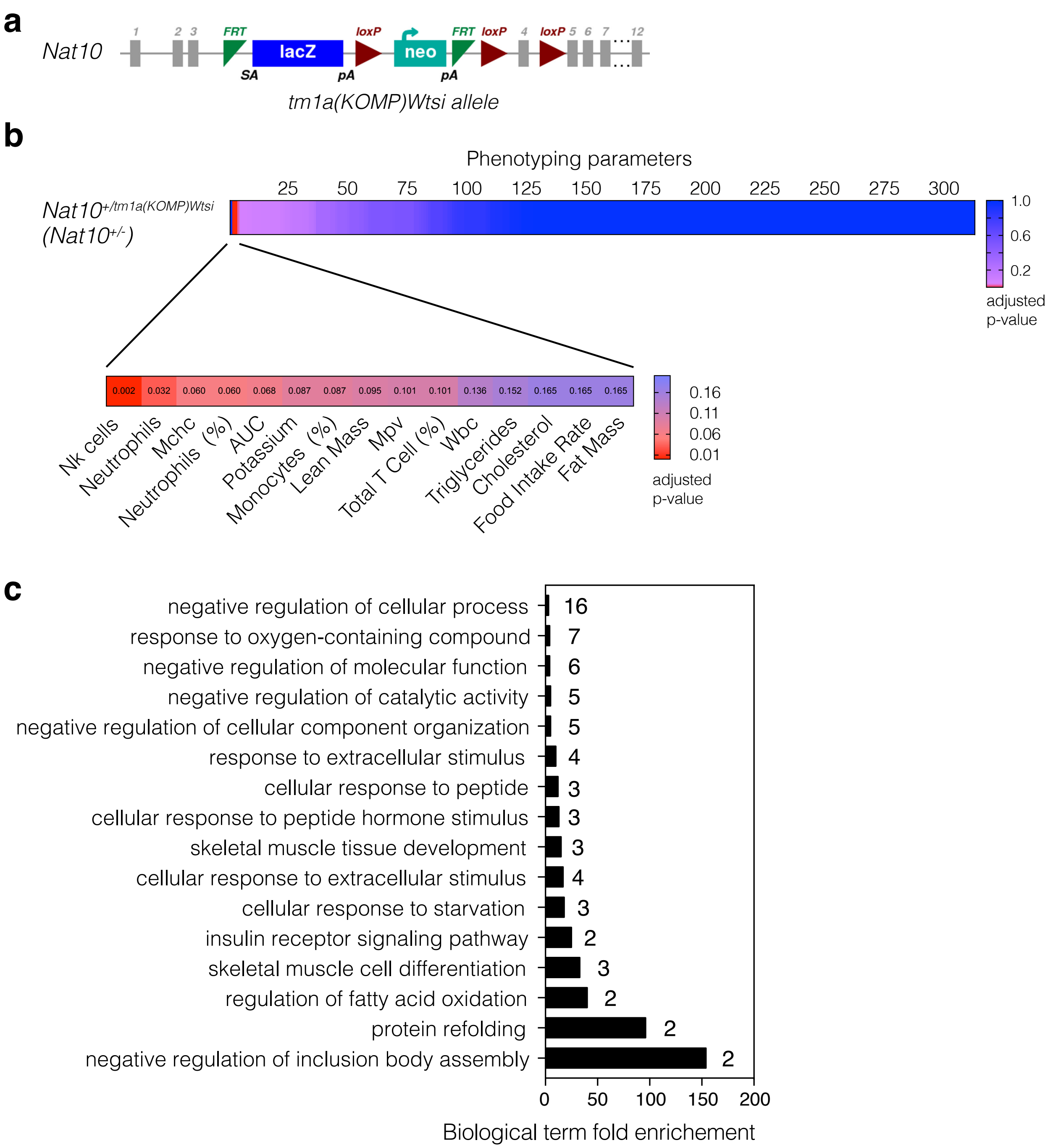

Supplementary Fig. 3: Engineering and characterisation of a *Nat10*$^{+/-}$ mouse model. **a** Schematic representation of the gene-trap construct engineered to disrupt the *Nat10* gene. **b** The high throughput phenotyping pipeline had n≥337 WT female mice, n≥337 WT male mice, n≥6 *Nat10*$^{+/-}$ female mice and n≥6 *Nat10*$^{+/-}$ male mice. The exact n number depended on the screen and can be seen in the respective excel data files (Supplementary Data 1). Top: heat-map of adjusted p-value of the phenotypic outcomes of the represented assays in *Nat10*$^{+/-}$ mice compared to WT (red: significant parameter; blue: non-significant parameter). A significant difference in Klrg1 mature natural killer (Nk) cell number (p^a=0.002), Nk cell number (p^a=0.002) and neutrophil number (p^a=0.032) was

observed specifically in the *Nat10*$^{+/-}$ males as compared to WT males, but not in females. Raw data are presented in Supplementary Data 1. Bottom: zoom-in on significant parameters from the heat-map; Mchc (mean corpuscular hemoglobin concentration); AUC (weight area under curve); Mpv (mean platelet volume); Wbc (white blood cells). **c** Gene ontology analysis showing top scoring terms fold enrichment (false discovery rate: FDR<0.03; p<0.0005) from genes shown in Fig. 3**d**. The number of target genes in each category is shown.

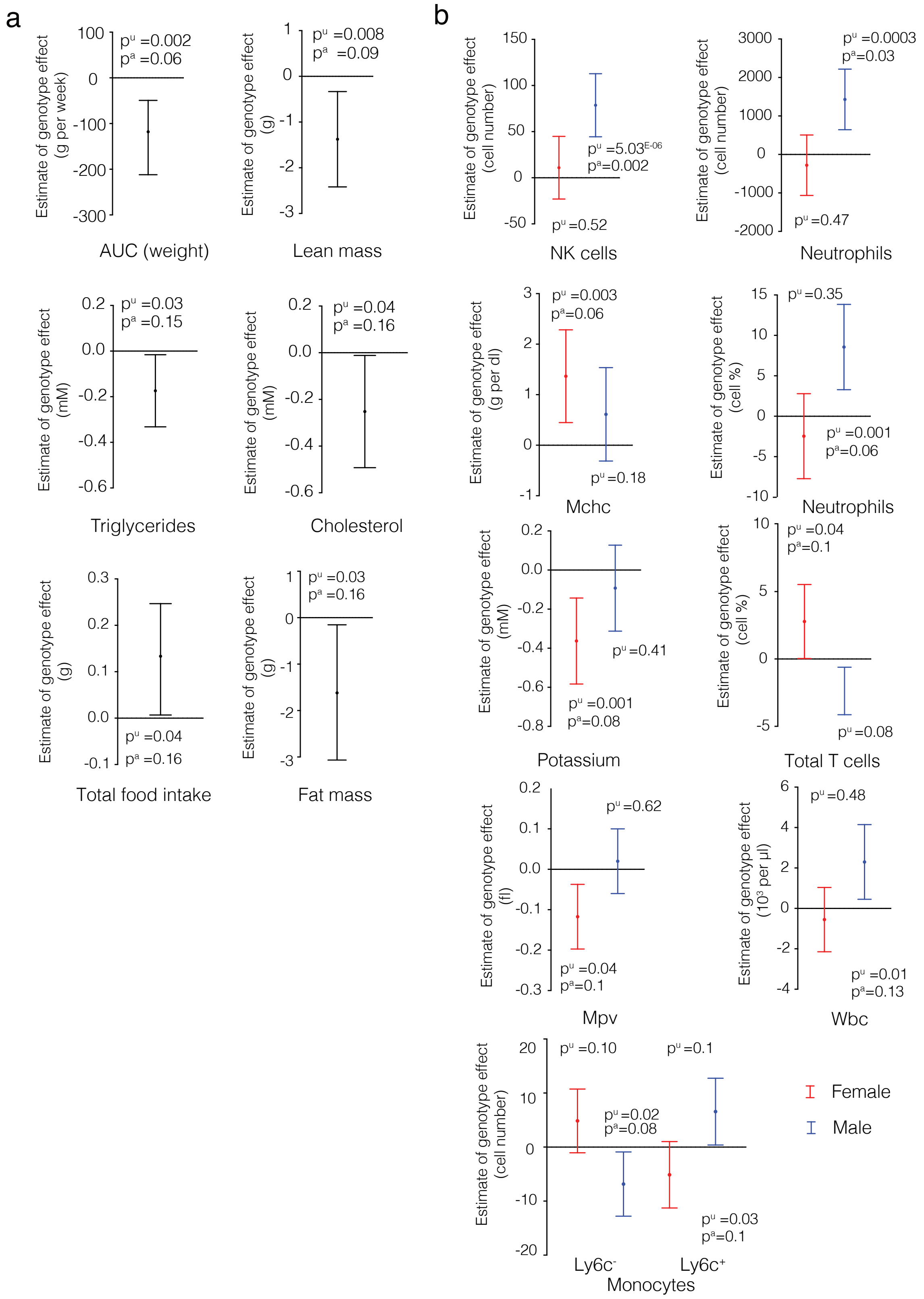

Supplementary Fig. 4: Significant parameters from the *Nat10*$^{+/-}$ phenotypic heat-map. From the mixed-model analysis, the estimated *Nat10*$^{+/-}$ genotype effect with 95%

confidence intervals is shown for significant parameters. Unadjusted p values (p^u) and adjusted p-values (p^a) are represented. The high throughput phenotyping pipeline had n≥337 WT female mice, n≥337 WT male mice, n≥6 *Nat10*$^{+/-}$ female mice and n≥6 *Nat10*$^{+/-}$ male mice. The exact n number depended on the screen and can be seen in the respective excel data files (Supplementary Data 1). **a** Some variables including weight area under the curve (AUC), lean mass, triglycerides, cholesterol, total food intake and fat mass showed differences in both males and females. **b** Other variables including natural killer (NK) cells, neutrophils (number and percentage), mean corpuscular hemoglobin concentration (Mchc), potassium, monocytes comprising Ly6c negative (Ly6c$^-$) and Ly6c positive (Ly6c$^+$), mean platelet volume (Mpv), number of total T cells and white blood cells (Wbc) showed sexual dimorphism.

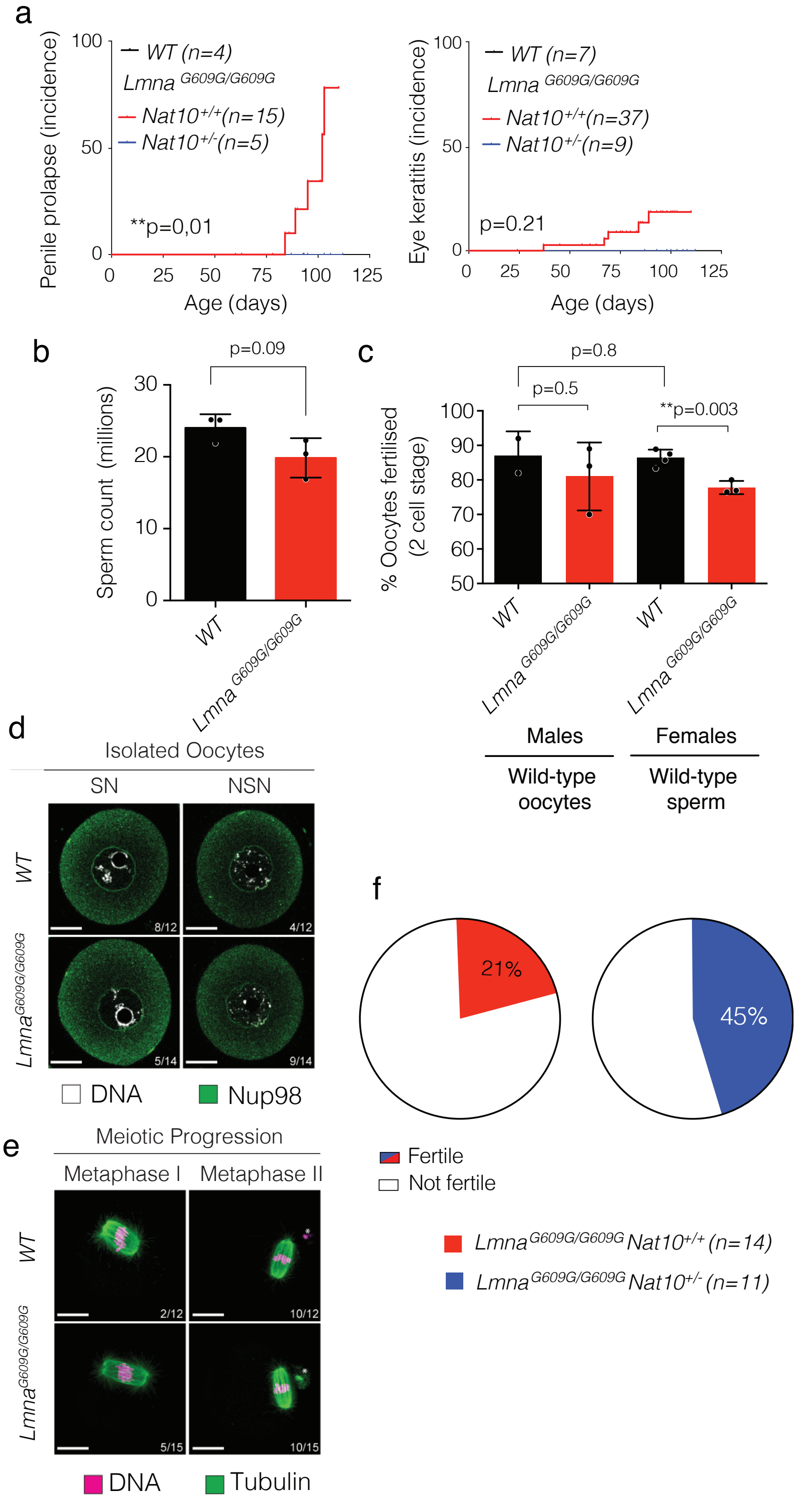

Supplementary Fig. 5: *Lmna*$^{G609G/G609G}$ *Nat10*$^{+/-}$ mice display fitness enhancement. a Graphs representing the incidence of male penile prolapse (left) and eye keratitis (right) in WT as compared to *Lmna*$^{G609G/G609G}$ single mutants and *Lmna*$^{G609G/G609G}$ *Nat10*$^{+/-}$

double mutant mice. Incidence based on phenotype being indicated by mouse facility technicians and validated by the facility named veterinary surgeon. For penile prolapse Kaplan–Meier graphs (Log-rank Mantel-Cox test; Chi-square 8.415; df=2; *p-value=0.01). For eye keratitis Kaplan–Meier graphs (Log-rank Mantel-Cox test; Chi-square 3.111; df=2; p-value=0.21; ns=not significant). **b** Quantification of sperm cells in WT (n=3) and *Lmna*$^{G609G/G609G}$ (n=3) mice, showing no significant difference (p=0.09; two-tailed Student's t-test). Bar graphs representing mean ± s.d.. **c** Quantification of *in vitro* fertilisation potential of sperm from WT (n=2) and *Lmna*$^{G609G/G609G}$ (n=3) male mice using oocytes from WT mice and of oocytes from WT (n=4) and *Lmna*$^{G609G/G609G}$ (n=3) female mice using sperm from WT mice. Bar graphs representing mean ± s.d.. Statistical significance was calculated using two-tailed Student's t-test. **d** and **e** Oocytes isolated from WT and *Lmna*$^{G609G/G609G}$ female mice are normal and show normal meiotic progression. Representative images show oocytes isolated from 8-week old WT and *Lmna*$^{G609G/G609G}$ female mice. The number of oocytes for each category is shown in bottom right-hand corner. Asterisk shows polar body. Scale bars, 20 µm. **f** NAT10 inhibition appeared to increase fertility in *Lmna*$^{G609G/G609G}$ mice but was not statistically significant due to low number of mice (p=0.29; two-tailed Fisher exact test).

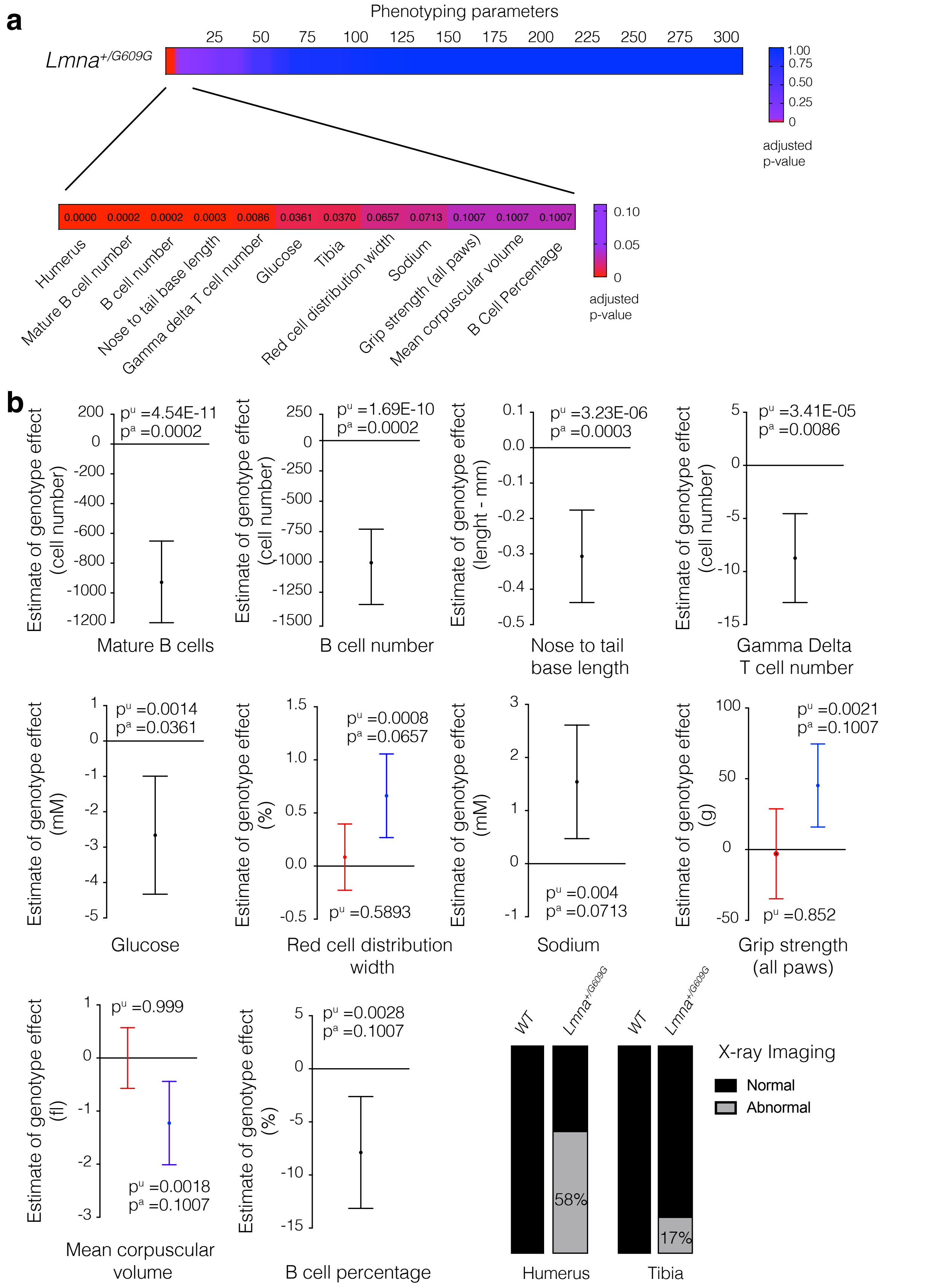

Supplementary Fig. 6: Phenotypic characterisation of *Lmna*$^{+/G609G}$ mice. The high

throughput phenotyping pipeline had n≥300 WT female mice, n≥300 WT male mice, n≥5 *Lmna*$^{+/G609G}$ female mice and n≥5 *Lmna*$^{+/G609G}$ male mice. The exact n number depended on the screen and can be seen in the respective excel data files (Supplementary Data 4). **a** Heat-map of adjusted p-values of the phenotypic outcomes from the high throughput phenotyping of in *Lmna*$^{+/G609G}$ mice compared to WT (red: significant parameter; blue: non-significant parameter). Raw data and statistical output are presented in Supplementary Data 4. A zoom-in on significant parameters from the heat-map is shown. **b** From the mixed-model analysis**,** the estimated *Lmna*$^{+/G609G}$ genotype effect with 95% confidence intervals is shown for significant variables. Unadjusted p value (p^u) and adjusted p-values (p^a) are represented. Some variables including red cell distribution width, grip strength (all paws) and mean corpuscular volume showed sexual dimorphism (females-red bars; males blue bars). For the categorical parameters identified as significant using the Fisher exact test, such as humerus and tibia, X-ray imaging was used to distinguish between normal and abnormal morphology shown as a proportion plot.

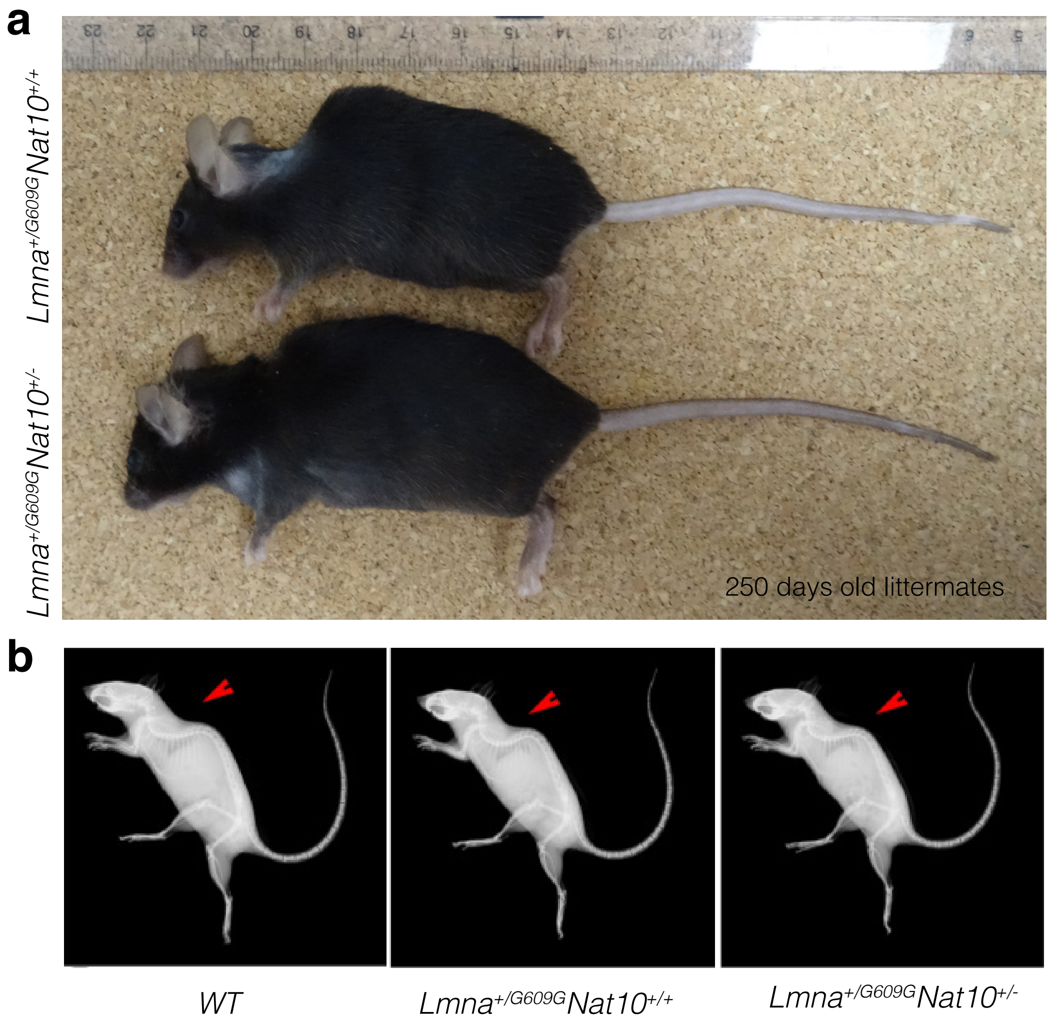

Supplementary Fig. 7: *Nat10* depletion reduces back curvature in *Lmna*$^{+/G609G}$ mice. **a** Images of two littermates at 250 days old. The *Lmna*$^{+/G609G}$ mouse displayed strong back curvature that was not observed in the *Lmna*$^{+/G609G}$*Nat10*$^{+/-}$ mouse. **b** X-ray of the indicated mouse genotypes. Arrows indicate back curvature, and show that in the heterozygous *Lmna*$^{+/G609G}$ mice, it is corrected by *Nat10* depletion. For WT mice, the occurrence of back curvature was rare, while we always see the phenotype in *Lmna*$^{+/G609G}$*Nat10*$^{+/+}$ *(n=30)* and delayed in *Lmna*$^{+/G609G}$ *Nat10*$^{+/-}$*(n=13*).

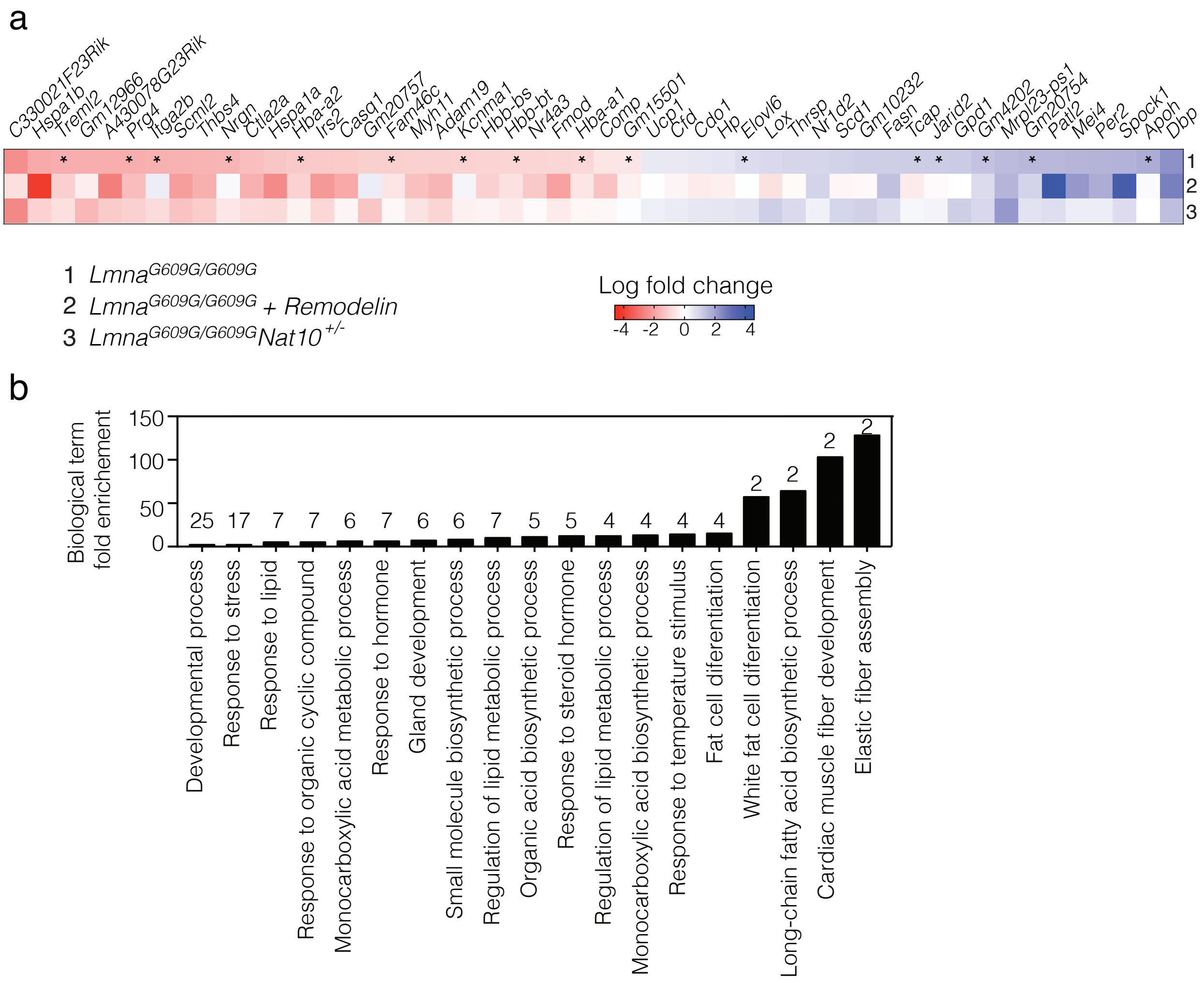

Supplementary Fig. 8: Identification of signatures for *Nat10* inhibition in cells and *in vivo*. **a** Heat map of genes differentially expressed in the heart of *Lmna*$^{G609G/G609G}$ mice (n=2) compared to wild-type (lane 1; n=2) and upon *Nat10* reduction (lane 2; n=2) or Remodelin treatment (lane 3; n=2). Asterisks indicate genes showing same expression level trends in both *Lmna*$^{G609G/G609G}$*Nat10*$^{+/-}$ and *Lmna*$^{G609G/G609G}$ Remodelin compared to *Lmna*$^{G609G/G609G}$. **b** Biological term analysis showing top-scoring terms fold-enrichment (FDR<0.03; p<0.0005) from the genes in row 1 of E. The number of target genes in each category is shown.

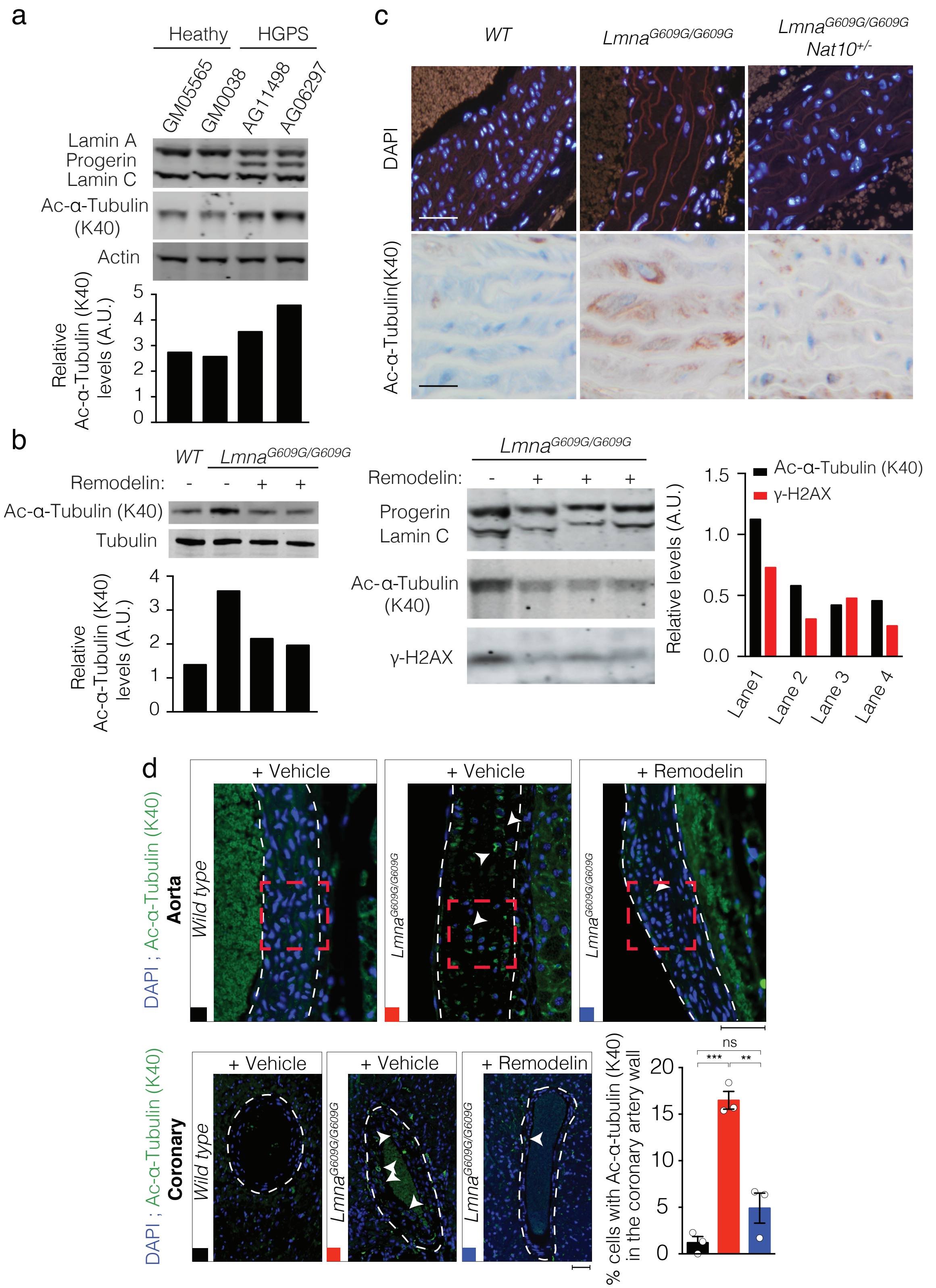

Supplementary Fig. 9. *Lmna*$^{G609G/G609G}$ mice display increased Lys-40 tubulin acetylation that is reduced upon NAT10 inhibition. a Representative western blot showing high tubulin acetylation levels in 2 different HGPS patient cell lines compared to

two healthy controls. Western blots were repeated on independent samples ($n \geq 3$) **b** Representative western blot showing that NAT10 inhibition reverses the high tubulin acetylation levels in heart tissues from indicated genotypes and that decreased tubulin acetylation correlates with decreased gamma-H2AX levels. Western blots were repeated more than one time on multiple independent samples ($n \geq 3$). **c** NAT10 inhibition restores normal levels of acetyl-tubulin, as observed by immunohistochemistry in heart sections of *Lmna*$^{G609G/G609G}$ mice; scale bars are 40μM and 50μM respectively. All the immunohistochemistry experiments were performed on independent mice ($n \geq 3$/genotype). **d** Representative immunofluorescence images of acetyl-tubulin (K40) in aortas (size bar 100μm) and coronary arteries (size bar 50μm) of terminal mice of the indicated genotypes and treatment (see also Supplementary Table 4); dotted white line delineates the aorta and the coronary respectively. Higher magnification snap-shots (red dotted squares) of the aorta are presented alongside quantification in Fig. 5**d**. Lys-40 (K40) α-tubulin acetylation (green; white arrowheads point to cells that show increased K40-acetylation) is increased in the *Lmna*$^{G609G/G609G}$ mice that and significantly decreased upon Remodelin treatment (n=3 individual mice; mean ± s.d.; individual data points represented; ns = not significant, *$p<0.05$; **$p<0.01$, ***$p<0.001$; two-tailed Student's t-test).

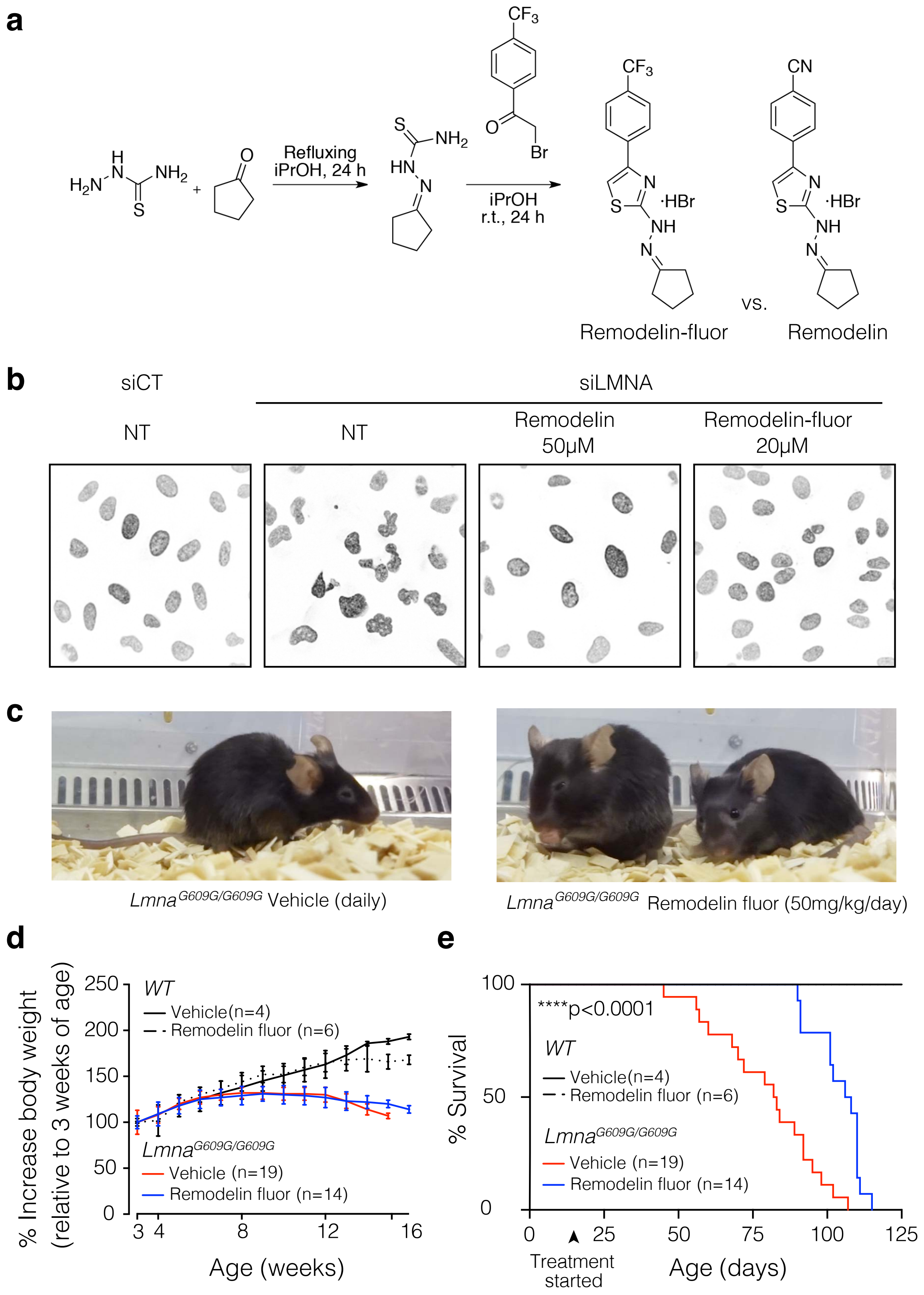

Supplementary Fig. 10: Identification of a more potent Remodelin analogue. a Synthesis and chemical structure of Remodelin-fluor (left) and its analogue Remodelin

(right). **b** Representative images of U2OS cells transfected with siRNA control (siCT) or siRNA against Lamin A/C (siLMNA) and treated for 24h. Nuclear shape was assessed by microscopy following DAPI staining of the nucleus. Remodelin-fluor showed increased potency in rescuing nuclear shape defects of Lamin A/C depleted cells (siLMNA) compared to Remodelin, and relative to the mock-treated cells (NT). **c** Representative images of mice treated with Remodelin-fluor. Long-term treatment of Remodelin-fluor at 50mg per kg per day caused hair greying, specifically around the eyes, in all treated mice. **d** No drug dependent body weight loss or toxicity was observed upon 50mg per kg per day of Remodelin-fluor treatment. Bars represent mean ± s.d.. **e** 50mg per kg per day oral Remodelin-fluor delivery led to a 30% Kaplan–Meier area under the curve increase age at endpoint (20% body weight loss) in the *Lmna*$^{G609G/G609G}$ mice (see Supplementary Table 1). Data for the vehicle-treated *Lmna*$^{G609G/G609G}$ mice (red line) was combined across experiments to allow comparison of age at endpoint and is also presented in Fig. 1**e**. ****Log-rank (Mantel-Cox) test comparison between *Lmna*$^{G609G/G609G}$ treated with Vehicle and *Lmna*$^{G609G/G609G}$ treated with Remodelin-fluor.

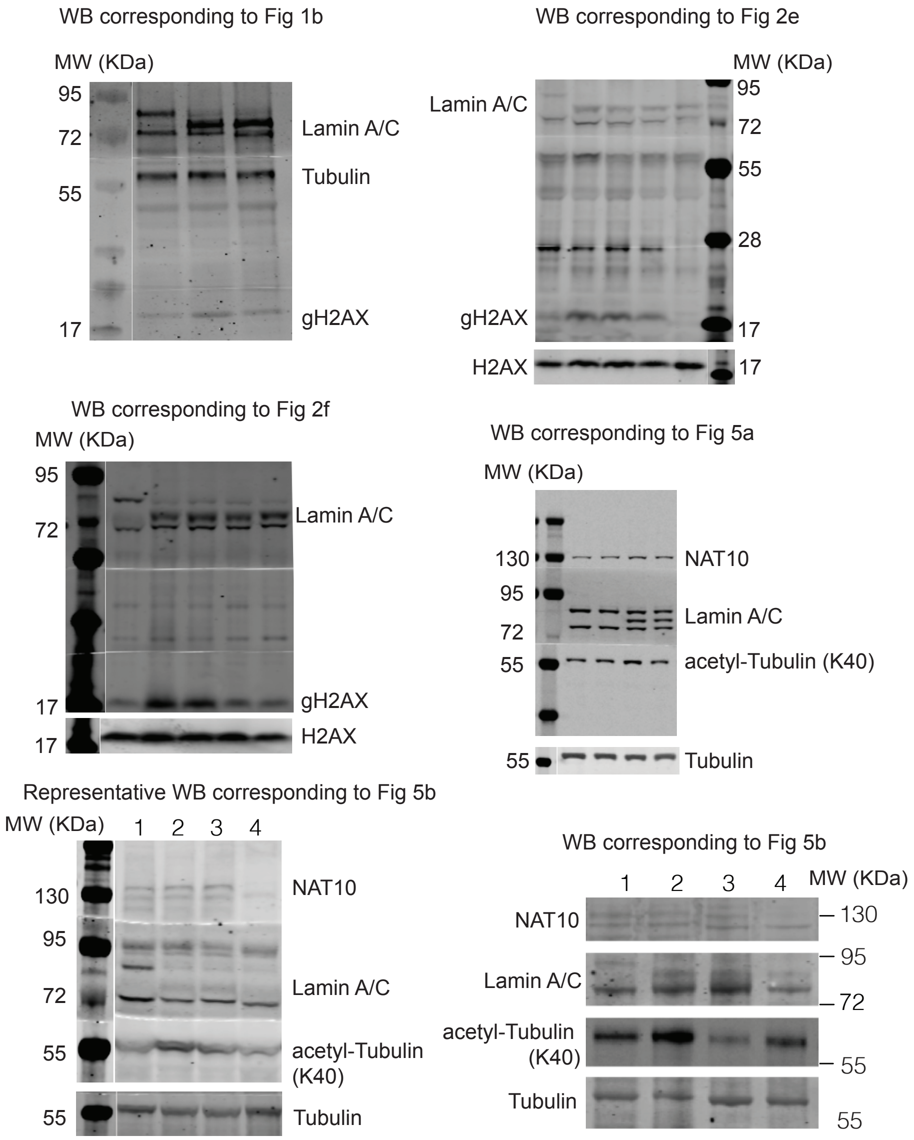

Supplementary Fig. 11: Uncropped blots corresponding to the main figures. Each panel represents one individual western blot as shown in the corresponding indicated main figure. Membranes from the WB corresponding to Figure 5b were scanned separately (bottom right), we therefore provide here another representative uncropped WB (bottom left) obtained from another set of mice.

Supplementary Tables

Groups	n	Treatment	Dose (mg/kg)	Dosing Route	Schedule	Sample Collection			
						Plasma (50µl)	Whole Blood	Heart	Muscle
1	3 Female	Vehicle	100	PO	Single dose	15min, 30min, 1h	NA	1h*	1h
2	3 Female	Remodelin	100	PO		15min, 30min, 1h	NA	1h	1h
3	3 Female	Vehicle	100	SC		15min, 30min, 1h	NA	1h	1h
4	3 Female	Remodelin	100	SC		15min, 30min, 1h	NA	1h	1h

Note: 1. n: animal number;
1. Dosing volume: adjust dosing volume based on body weight, SC (sub-cutaneous) group 10 ml/kg; PO (oral) group 10 ml
* One hour after treatment

Supplementary Table 1. Design table for the administration of the test articles and sample collection in each study group.

Groups	n	Treatment	Dose (mg/kg)	Dosing Route	Schedule	Sample Collection				Fasting
						Plasma (50μl)	Whole Blood	Serum	Tissue	
1	3 male	Remodelin	1	IV	Single dose	5min, 2h	NA	NA	NA	Over night before the last dose
2	3 male		1	IV		15min, 4h,	NA	NA	NA	Over night before the last dose
3	3 male		1	IV		30min,8h,	NA	NA	NA	Over night before the last dose
4	3 male		1	IV		1h, 6h,24h	NA	NA	NA	Over night before the last dose
5	3 male		5	P.O.		15min, 4h	NA	NA	NA	Over night before the last dose
6	3 male		5	P.O.		30min, 6h,	NA	NA	NA	Over night before the last dose
7	3 male		5	P.O.		1h,8h,	NA	NA	NA	Over night before the last dose
8	3 male		5	P.O.		2h, 24h	NA	NA	NA	Over night before the last dose
9	5 male		NA	NA		As much as possible	NA	NA	NA	Blank control

Note: 1. n: animal number;
1. Dosing volume: adjust dosing volume based on body weight, IV (intra-venous) group ; PO (oral) group

Supplementary Table 2. Pharmacokinetics Evaluation of Remodelin *via* IV and PO administration.

Genotype comparisons	# deaths (censored)	Median survival (days)	Area Under the Curve (AUC)	% AUC	Log-rank Mantel-Cox test; Chi-square	df	P value
Lmna$^{G609G/G609G}$ *No treatment*	33 (0)	83.0	8015	100%	0.32	1	ns; 0.56
Lmna$^{G609G/G609G}$ *Vector*	18 (0)	82.5	7644	95%			
Lmna$^{G609G/G609G}$ *Vector*	18 (0)	82.5	7644	100%	5.99	1	*; <0.014
Lmna$^{G609G/G609G}$ *Remodelin*	12 (1*)	97.0	9575	125%			
Lmna$^{G609G/G609G}$ *Vector*	18 (0)	82.5	7644	100%	17.6	1	****; <0.0001
Lmna$^{G609G/G609G}$ *Remodelin Fluor*	15 (0)	107.0	9929	130%			
Lmna$^{G609G/G609G}$ *Nat10*$^{+/+}$	34 (0)	85.0	8200	100%	7.82	1	**; <0.052
Lmna$^{G609G/G609G}$ *Nat10*$^{+/-}$	15 (1**)	103.0	9577	117%			
Lmna$^{+/G609G}$ *Nat10*$^{+/+}$	30 (0)	284.5	27778	100%	4.98	1	*; <0.025
Lmna$^{+/G609G}$ *Nat10*$^{+/-}$	13 (0)	333.0	30246	109%			

* this mouse was within normal weigh and was censored due to a health concern reported by the room technician as abnormal mass at 79 days of age; upon necropsy a hemangioma was found
** this mouse was within normal weigh and was censored due to a health concern reported by the room technician as swolen abdomen at 32 days of age; upon necropsy no abnormalities were found

Supplementary Table 3. Comparison of survival rates of mice in this study based on Kaplan-Meyer analysis

Mouse name	Genotye	Treatment	Age (days)
LMNA16.1f	*Lmna* $^{+/+}$	Vector	82*
LMNA39.2g	*Lmna* $^{+/+}$	Vector	126
LMNA40.2b	*Lmna* $^{+/+}$	Vector	125
LMNA30.2a	*Lmna* $^{+/+}$	Remodelin	133
LMNA33.2c	*Lmna* $^{+/+}$	Remodelin	133
LMNA39.1g	*Lmna* $^{+/+}$	Remodelin	148
LMNA16.1b	*Lmna* $^{G609G/G609G}$	Vector	82
LMNA27.1e	*Lmna* $^{G609G/G609G}$	Vector	68
LMNA30.2b	*Lmna* $^{G609G/G609G}$	Vector	107
LMNA32.1b	*Lmna* $^{G609G/G609G}$	Vector	57
LMNA39.2h	*Lmna* $^{G609G/G609G}$	Vector	92
LMNA39.2i	*Lmna* $^{G609G/G609G}$	Vector	92
LMNA30.2f	*Lmna* $^{G609G/G609G}$	Remodelin	112
LMNA40.2d	*Lmna* $^{G609G/G609G}$	Remodelin	96
LMNA37.1a	*Lmna* $^{G609G/G609G}$	Remodelin	113
LMNA39.1e	*Lmna* $^{G609G/G609G}$	Remodelin	82
LMNA39.2a	*Lmna* $^{G609G/G609G}$	Remodelin	89
LMNA62.1i	*Lmna* $^{G609G/G609G}$	Remodelin	98

* This mouse was culled as littermate-control with one of the Vector HGPS mice thus was censored in the survival curve; the WT mice have never lost 20% body-weight and have been terminated after the last HGPS mouse was culled

Supplementary Table 4. List of mice used for pathologic assessment
