# Supplementary material for: Targeting of NAT10 enhances healthspan in a mouse model of human accelerated aging syndrome
Source: Nat Commun. 2018 Apr 27;9:1700. doi: 10.1038/s41467-018-03770-3 (PMC5923383; doi:10.1038/s41467-018-03770-3)
Supplement: Supplementary file 3 — Description of Additional Supplementary Files [file 41467_2018_3770_MOESM3_ESM.pdf]

## Description of Additional Supplementary Files

**Supplementary Movie 1:** *Lmna*$^{G609G/G609G}$ terminal mouse (20% body weight loss; 13-14 weeks old) showing premature ageing. The mouse presents rough hair coat, dirty incision, squinted eyes, hunched walk and moderate agitation, slight dehydration, pruritic and restless.

**Supplementary Movie 2:** *Lmna*$^{G609G/G609G}$ and *Lmna*$^{G609G/G609G}$*Nat10*$^{+/-}$ age matched littermate controls at 13 weeks of age. *Lmna*$^{G609G/G609G}$*Nat10*$^{+/-}$ mouse (no weight loss; not terminal; right side at the beginning of the movie) is fitter than *Lmna*$^{G609G/G609G}$ mouse (20% body weight loss – terminal; left side at the beginning of the movie). While the *Lmna*$^{G609G/G609G}$ mouse is not well groomed, has an awkward gait, is slightly hunched and moves slowly, the *Lmna*$^{G609G/G609G}$*Nat10*$^{+/-}$ mouse is normal, well groomed, alert; active; in good condition and shows normal behaviour.

**Supplementary Movie 3:** Terminal *Lmna*$^{G609G/G609G}$*Nat10*$^{+/-}$ mice compared to WT littermate control. The *Lmna*$^{G609G/G609G}$*Nat10*$^{+/-}$ mice (two smaller mice; 20% body weight loss – terminal; upper and lower left side at the beginning of the movie) at 15-16 weeks of age show no other phenotype than body weight loss as compared to WT littermate control (one mouse; lower right side at the beginning of the movie). All mice are well groomed, alert; active; in good condition and show normal behaviour.

**Supplementary Data file 1**: Phenotypic analysis of *Nat10*$^{+/-}$ mice including over 300 phenotypic parameters (categorical and non-categorical) and their associated statistical calling. This data is also freely available from IMPC (http://www.mousephenotype.org/).

**Supplementary Data file 2**: RNAseq analysis of heart tissues from *Nat10*$^{+/-}$ mice representing the statistically significant upregulated/downregulated genes, as called by 3 different programs (deseq2, edgeR and cuffdiff) as well as VLAD pathway analysis of significantly enriched GO terms. Raw data is freely available from GEO public repository (https://www.ncbi.nlm.nih.gov/geo/).

**Supplementary Data file 3:** Phenotypic analysis of *Lmna*$^{G609G/G609G}$*Nat10*$^{+/-}$ and *Lmna*$^{+/G609G}$ *Nat10*$^{+/-}$ mice at 9-12 weeks including over 300 phenotypic parameters (categorical and non-categorical) and associated statistical calling. This data is also freely available from Zenodo (https://www.zenodo.org).

**Supplementary Data file 4:** Phenotypic analysis of *Lmna*$^{+/G609G}$ mice including over 300 phenotypic parameters (categorical and non-categorical) and associated statistical calling. This data is also freely available from Zenodo (https://www.zenodo.org).

**Supplementary Data file 5:** RNAseq analysis of heart tissues from *Lmna*$^{G609G/G609G}$*Nat10*$^{+/-}$ mice and Remodelin treated *Lmna*$^{G609G/G609G}$ mice representing the statistically significant upregulated/downregulated genes, as called by 3 different programs (deseq2, edgeR and cuffdiff) as well as VLAD pathway analysis of significant enriched GO terms. Raw data is freely available from GEO public repository (https://www.ncbi.nlm.nih.gov/geo/).
