# Supplementary figures and images for: Publisher Correction: Targeting of NAT10 enhances healthspan in a mouse model of human accelerated aging syndrome
Source: Nat Commun. 2026 Mar 18;17:2537. doi: 10.1038/s41467-026-69133-5 (PMC13000205; doi:10.1038/s41467-026-69133-5)

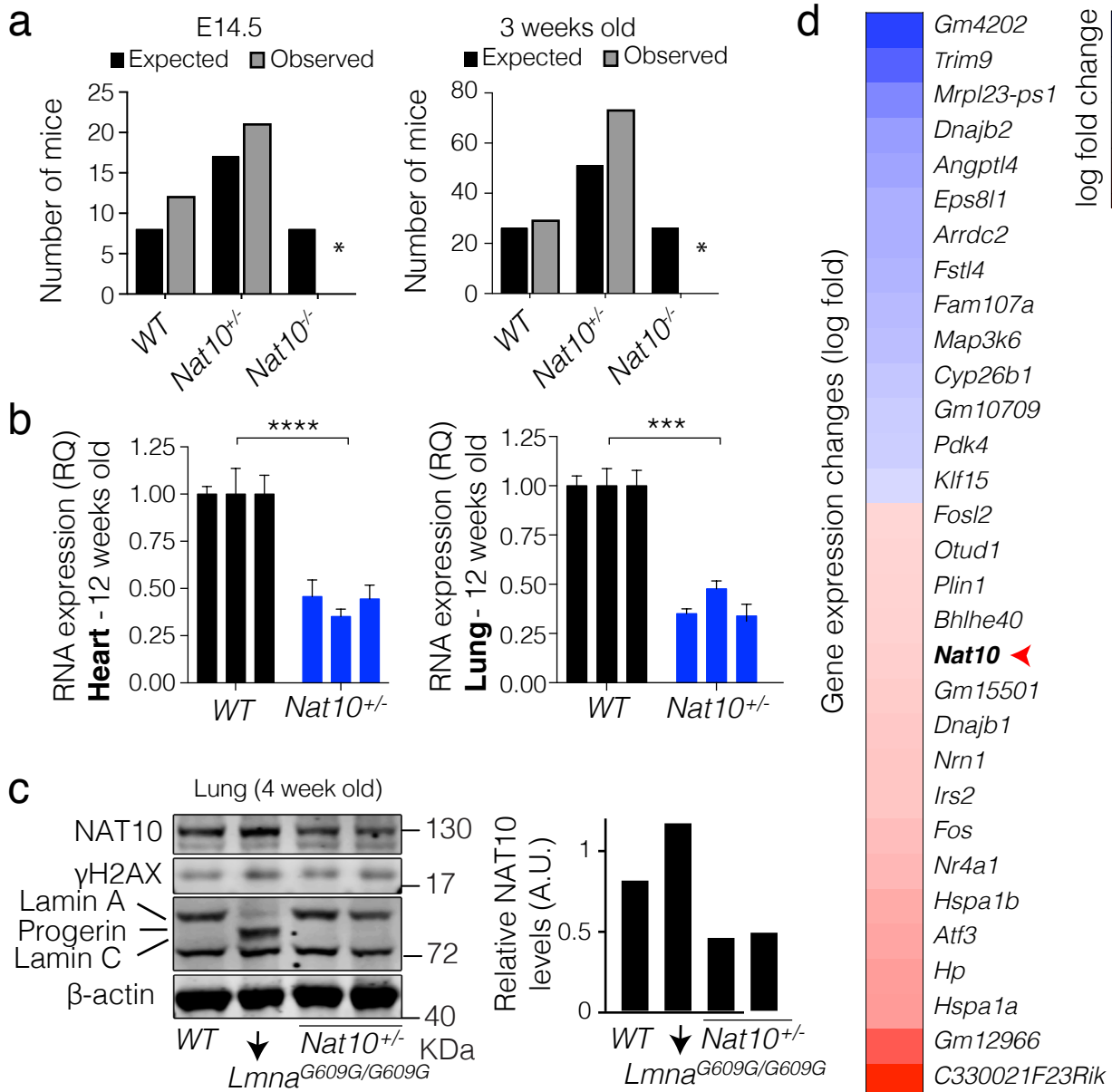

Supplement: Supplementary file 1 — Correct Fig. 3. [file 41467_2026_69133_MOESM1_ESM.pdf]
